# Evaluation of the implementation of an intervention aimed at expanding Covid-19 testing, case telemonitoring and surveillance actions in Primary Health Care

**DOI:** 10.11606/s1518-8787.2026060006688

**Published:** 2026-04-17

**Authors:** Gerluce Alves Pontes da Silva, Thais Regis Aranha Rossi, Sisse Figueiredo de Santana, Alcione Brasileiro Oliveira, Ana Maria Freire de Souza Lima, Trícia Silva Santos, Izabel Cristina Neves Ramos, Carolina Pedroza de Carvalho Garcia, Laio Magno, Inês Dourado, Lígia Maria Vieira da Silva

**Affiliations:** I Universidade Federal da Bahia. Instituto de Saúde Coletiva. Salvador, BA, Brasil; II Universidade do Estado da Bahia. Departamento de Ciências da Vida. Salvador, BA, Brasil; III Universidade Federal da Bahia. Faculdade de Odontologia. Departamento de Odontologia Social e Pediátrica. Salvador, BA, Brasil; IV Secretaria Municipal de Saúde de Camaçari. Camaçari, BA, Brasil; V Fundação Oswaldo Cruz. Instituto Gonçalo Moniz. Salvador, BA, Brasil

**Keywords:** Evaluation in Health, Health Surveillance, Primary Health Care

## Abstract

**OBJECTIVE:**

To evaluate the degree of implementation of an intervention aimed at expanding testing, isolation, quarantine, and telemonitoring of Covid-19 cases (TQT-Covid-Strategy) in primary healthcare units within an administrative health region of a municipality in Northeastern Brazil.

**METHODS:**

A decision matrix was used with dimensions and criteria derived from a logical model developed based on literature review and technical guidelines of the World Health Organization, the Brazilian Ministry of Health, and the host municipality of the administrative health region. Interviews were conducted with 121 key informants.

**RESULTS:**

The degree of implementation in the 17 health units surveyed (12 Family Health Units and five health centers) varied according to the final classification, dimensions, and criteria. Overall, most units were considered as having the intervention partially implemented, with the highest rated dimension being "accessibility to testing," implemented in 82.3% of the units, followed by the use of the "digital platform," partially implemented in 15 units. The dimension with the lowest score percentage was "monitoring cases and surveillance strategies".

**CONCLUSIONS:**

The intervention led to significant improvements in access to testing, supported by the adoption of communication strategies that enhanced the population's adherence. Contextual, operational, technical, and acceptance factors among health professionals and users represented important challenges to implementation. To ensure sustainability and dissemination of this strategy, investments are required to strengthen Primary Health Care, including improvement in infrastructure, expansion of the health workforce, and professionals training.

## INTRODUCTION

The Covid-19 pandemic has imposed a global health crisis, generating complex challenges for health systems around the world^1^. The initial management of this crisis evidenced the difficulty in translating global or national health policies into local contexts, often with greater emphasis on hospital care to the expense of health surveillance^2^.

In response, countries with successful experiences expanded access to tests, isolation, contact tracing, and telemonitoring^3-6^. Primary Health Care (PHC) has played an important role in this scenario^7-9^, although it was significantly impacted by changes related to the organization of services, the health of professionals, in addition to care types^10-13^. In Brazil, PHC teams fundamentally acted in surveillance and provision of care, even in the face of structural challenges^13-15^.

Given the need to strengthen the response to the pandemic in PHC, especially in vulnerable territories, the *Estratégia-TQT-Covid* [TQT-Covid-Strategy] was proposed aiming at expanding testing for Covid-19, isolation, quarantine, and telemonitoring of cases in PHC units. However, the mere existence of an intervention does not guarantee its routine use; it is essential to understand how to implement it, the barriers encountered, and the facilitating factors involved^16^.

For the successful implementation of public health measures during the pandemic, the availability of resources (equipment, personnel, financial) was essential^17^. The reorganization of team work processes to integrate surveillance and care for cases also represented a challenge^18^. Conversely, popular adherence acted as a key facilitator, contributing for the success of the measures^5,6^. In order to systematize successful experiences and the problems faced, researchers highlight the importance of integrating evaluation into the design and implementation of public health interventions and policies^19^. This implies the need to develop specific capacities to evaluate such interventions.

The multicenter research project TQT-Covid-Strategy was developed in a capital in Northeastern Brazil. This initiative had the participation of four research institutions, the support of the Brazilian Ministry of Health, and was funded by an international agency (Unitaid)^20^.

The intervention was implemented between July 2022 and February 2023, focusing on challenges^21^ identified in coping with the pandemic and based on a situation analysis. These challenges included concentration of testing in some health units, lack of early identification of cases and contact tracing, lack or low performance of telemonitoring of cases, lack of communication strategies on Covid-19 in the territories, difficulty in accessing test reports, inadequate infrastructure of Health Centers (*unidades básicas de saúde* – UBSs) and insufficient health professionals, difficulties in recording actions, lack of adequate electronic devices and unstable Internet connection, manual completion of notification forms, in addition to the lack of training provision for PHC professionals^21^.

Seeking to evaluate the implementation of the TQT-Covid-Strategy, in this study, we aimed to determine the extent to which it was implemented as planned in health units of a specific administrative region of a municipality in Northeastern Brazil and to identify the elements that favored or hindered this process.

## METHODS

A study was conducted to assess the degree of implementation of the TQT-Covid-Strategy in an Administrative Health Region (AHR), with coverage of about 400 thousand inhabitants, of a municipality in the Northeast Brazil. All UBSs of a health district were included. This district was selected due to its high social vulnerability, its registration of one of the highest numbers of Covid-19 cases at the beginning of the pandemic, and for being an area with strong teaching-care integration linked to one of the collaborating research institutions.

As a criterion for selecting the units within this district, the presence of permanent professionals in their staff, with at least one physician, was required. Thus, 17 units were selected, comprising 12 Family Health Units (FHU) and five UBSs. A project planning and implementation committee was created with 16 managers and professionals from the Municipal Department of Health (*Secretaria Municipal de Saúde* – SMS) and AHR as well as researchers. This committee met weekly and actively participated in the planning and implementation process throughout the project.

To develop the evaluative research, initially, the logical model of the intervention was developed, with components, required actions, and intermediate and final results of the intervention (Figure). Actions to promote the expansion of tests in the territory were part of the component "accessibility to testing." Regarding the "case monitoring and surveillance strategies," means for preventing the worsening of cases and control of the spread of the virus were included. A digital platform was structured and its use aimed to manage the activities carried out. For developing the model, evidence from scientific literature, international and national protocols validated by the World Health Organization, the Brazilian Ministry of Health, and the host municipality of the AHR were used.

**Figure:**
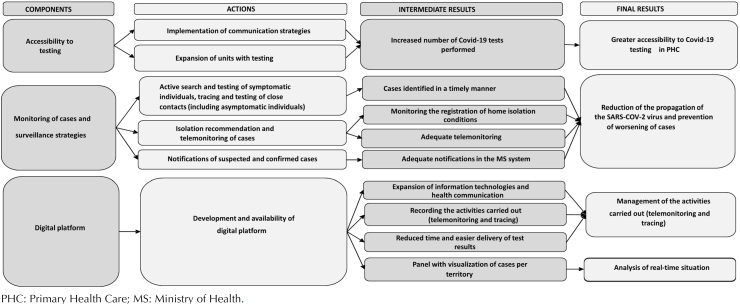
Logical model of TQT-Covid-Strategy.

Subsequently, a matrix was developed with dimensions, criteria, and standards for assessing the degree of implementation of the TQT-Covid-Strategy, submitted to a group of 20 specialists, composed of managers and professionals of SMS and AHR, as well as researchers for validation. Consensus was sought in relation to the proposed criteria and standards.

The final version of the matrix (Chart 1) presented 12 criteria deemed important and very important, with medium and high degrees of consensus. The maximum score that each health unit could achieve was fixed at 100 points, divided between the dimensions "accessibility to testing" (40 points), "case monitoring and surveillance strategies" (40 points), and "digital platform" (20 points).

**Chart 1:** Decision matrix for evaluating the degree of implementation of the TQT-Covid intervention.

Criteria	Standards for classification
1.1. Health communication strategies on prevention and testing (10 pts.).	1. Does not carry out health communication actions in units, territories, and social networks; 2. Carries out sporadic actions of health communication in units, territories, and social networks; 3. Carries out systematic actions of health communication in units, territories, and social networks.
1.2. Extended and continuous testing (30 pts.).	1. Testing interruptions; 2. Continuous testing only for symptomatic individuals of the registered territory; 3. Continuous testing of symptomatic individuals and contacts (symptomatic and asymptomatic) of the registered territory.
2.1. Telemonitoring of cases according to the protocol (five pts.).	1. Does not perform telemonitoring; 2. Performs it, but does not meet the deadline established by municipal protocols; 3. Performs it, meeting the deadline established by municipal protocols.
2.2. Percentage of telemonitored diagnosed users (10 pts.).	1. Does not perform telemonitoring; 2. < 80% of telemonitored diagnosed cases; 3. > 80% of telemonitored diagnosed cases.
2.3. Identification of contacts (five pts.).	1. Does not identify contacts of confirmed cases; 2. < 1 proportion of contacts identified from confirmed cases; 3. > 1 proportion of contacts identified from confirmed cases.
2.4. Contact tracing (10 pts.).	1. Contact tracing is not carried out; 2. Makes contact by telephone or other means of < 80% of the identified contacts; 3. Makes contact by telephone or other means of > 80% of the identified contacts.
2.5. Active search for symptomatic individuals in the territory (10 pts.).	1. Does not perform it; 2. Sporadically performs it; 3. Systematically performs it.
3.1. Use for real-time situation analysis (four pts.).	1. Does not use the digital platform for real-time situation analysis; 2. Sporadically uses the digital platform for real-time situation analysis; 3. The team systematically uses the digital platform for real-time situation analysis.
3.2. Use in access to test results (four pts.).	1. Does not use it, even with the users’ demand for medical report or the team's internal monitoring; 2. Sporadically uses it, even with users’ demand for medical report or the team's internal monitoring; 3. Systematically uses the digital platform to access test results due to demand for medical reports or the team's internal monitoring.
3.3. Use in telemonitoring (four pts.).	1. Does not use it; 2. Sporadically uses it to check the scheduling and alerts for telemonitoring; 3. Systematically uses it to check the scheduling and alerts for telemonitoring.
3.4. Percentage of confirmed cases registered on the platform and notified in e-SUS Notifica (four pts.).	1. Does not confirm notifications of confirmed cases; 2. Provides confirmation of up to 80% of notifications of confirmed cases; 3. Provides confirmation over 80% of notifications of confirmed cases.
3.5. Use in contact tracing (four pts.).	1. Does not use the tracing module to verify contacts to be traced; 2. Sporadically uses the tracing module to verify contacts to be traced; 3. Systematically uses the tracing module to verify contacts to be traced;

TQT-Covid: testing, isolation, quarantine, and telemonitoring of Covid-19 cases; e-SUS Notifica [e-SUS Notifies]: online reporting tool for notification of mild flu-like syndrome cases suspected and confirmed of the new coronavirus; pts.: points; 1: not implemented; 2: partially implemented; 3: implemented.

Data production covered analysis of the final report of the monitoring of the intervention's implementation; data from the TQT-Covid-System (digital platform developed for the intervention); and semi-structured interviews with 121 key informants, involving 15 managers, 53 health professionals, and 53 Community Health Agents (CHAs) of the selected units.

The interview script was developed according to the components of the logical model. Interviews were conducted in the very service between September and December 2022, being transcribed and encoded using the *NVivo* software. The score was based on triangulation of pieces of evidence. In other words, the evaluators considered and compared the information obtained from the different sources — the interviews with key informants (qualitative data and their interpretations), the information of the monitoring report, and the data of the digital platform (quantitative data and their metrics). Triangulation allowed a more robust assessment of the degree of implementation for each criterion, integrating the perception and experience of professionals with objective performance data.

To classify the degree of implementation of the TQT-Covid-Strategy, considering the decision matrix elaborated, the degree of implantation was classified in tertiles: not implemented (≥ 0 and < 33.3%), partially implemented (≥ 33.3% and < 66.6%), and implemented (≥ 66.6%). The degree of implementation was determined by the percentage (%) of points obtained in each component in relation to the proposed maximum score. The research was approved by the World Health Organization Research Ethics Committee, under protocol No. CERC.0128A and CERC.0128B.

## RESULTS AND DISCUSSION

### Assessment of the Degree of Implementation

The degree of implementation of the intervention in the 17 health units varied in terms of final classification, dimensions, and criteria (Chart 2). In the general classification, most of the units were classified as having the intervention partially implemented (10 FHUs and four UBSs), with the highest rated dimension being "accessibility to testing," implemented in 82.3% of the units (12 FHUs and two UBSs), followed by the use of the "digital platform," partially implemented in 15 units (11 FHUs and four UBSs). The dimension "monitoring of cases and surveillance strategies" obtained the lowest score, classified as not implemented in 76.5% of the units (nine FHUs and four UBSs). Pieces of evidence are synthesized in Table and Chart 3.

**Table:** Indicators of the decision matrix for assessing the degree of implementation of the TQT-Covid intervention in an administrative health region from a municipality in Northeastern Brazil (variation from July 2022 to March 2023).

Unit	Percentage of telemonitored diagnosed users		Proportion of contacts identified in relation to TR+ users		Percentage of traced contacts found		Percentage of confirmed cases registered on the platform and notified in the e-SUS Notifica		
	No. of telemonitored TR+ users (≥ 1 contact)	% of TR+ users with ≥ 1 contact	No. of contacts identified	Identified contacts/TR+ users	No. of traced contacts found	% of traced contacts found among those identified	No. of TR+ users	Number of TR+ cases registered on the platform with e-SUS Notifica ID	%
FHU 1	37	19.8	160	0.85	85	53.1	187	187	100
FHU 2	23	26.1	95	1.08	42	44.2	88	83	94.0
FHU 3	29	32.2	12	0.13	0	0	90	87	96.7
FHU 4	10	8.2	7	0.06	1	14.3	122	119	97.5
FHU 5	10	12.3	15	0.18	0	0	81	78	96.3
FHU 6	41	12.2	33	0.10	12	36.4	337	158	46.8
FHU 7	42	21.9	34	0.18	7	20.6	192	106	55.2
FHU 8	10	11.6	61	0.70	22	36.1	86	86	100
FHU 9	0	0	207	0.96	0	0	216	216	100
FHU 10	3	2.7	40	0.36	3	7.5	112	110	98.2
FHU 11	3	2.4	3	0.02	0	0	124	101	81.4
FHU 12	1	1.4	57	0.77	0	0	74	73	98.6
Total value FHU	209	12.2	724	0.42	172	23.7	1,709	1,404	82.1
UBS 1	5	13.2	5	0.13	1	20.0	38	32	84.0
UBS 2	8	17.4	3	0.06	0	0	46	43	93.5
UBS 3	49	16.4	14	0.05	5	35.7	298	298	100
UBS 4	2	2.9	1	0.01	0	0	70	70	100
UBS 5	49	31.6	110	0.71	53	48.2	155	150	96.8
Total value UBS	113	18.6	133	0.22	59	44.4	607	593	97.7
Total value units	322	13.9	857	0.37	231	27.0	2,316	1,997	86.2

TR+: Positive test; FHU: Family Health Unit; UBS: health center; e-SUS Notifica ID: identification number of the notification in the system.

**Chart 2:** Classification of the degree of implementation of the TQT-Covid intervention per Health Center in the administrative region of a Northeastern municipality.

Dimension	Criterion	Implementation status per unit																
		UBS 1	UBS 2	UBS 3	UBS 4	UBS 5	FHU 1	FHU 2	FHU 3	FHU 4	FHU 5	FHU 6	FHU 7	FHU 8	FHU 9	FHU 10	FHU 11	FHU 12
1. Accessibility to testing	1.1. Communication strategies on prevention and testing.	I	PI	PI	NI	PI	I	I	I	I	I	I	I	I	I	I	PI	PI
	1.2. Extended and continuous testing.	PI	PI	I	PI	I	I	I	I	I	I	I	I	PI	I	I	I	I
Dimension 1 status		I	PI	I	PI	I	I	I	I	I	I	I	I	PI	I	I	I	I
2. Monitoring of cases and surveillance strategies	2.1. Telemonitoring of positive cases according to the protocol.	PI	I	PI	NI	PI	I	PI	I	I	I	PI	I	I	NI	PI	PI	PI
	2.2. Percentage of telemonitored diagnosed users.	NI	NI	NI	NI	NI	NI	NI	NI	NI	NI	NI	NI	NI	NI	NI	NI	NI
	2.3. Identification of contacts.	NI	NI	NI	NI	I	I	I	NI	NI	NI	NI	NI	PI	I	NI	NI	I
	2.4. Contact tracing.	NI	NI	PI	NI	PI	PI	PI	NI	NI	NI	PI	NI	PI	NI	NI	NI	NI
	2.5. Active search for symptomatic individuals in the territory.	NA	NA	NA	NA	NA	NI	PI	NI	PI	NI	NI	NI	NI	NI	NI	NI	NI
Dimension 2 status		NI	NI	NI	NI	PI	PI	PI	NI	NI	NI	NI	NI	PI	NI	NI	NI	NI
3. Digital platform	3.1. Use for real-time situation analysis.	NI	I	NI	NI	NI	NI	NI	NI	NI	PI	PI	NI	NI	NI	PI	NI	NI
	3.2. Use in access to test results.	I	I	I	I	I	I	I	I	I	I	I	I	I	I	I	I	I
	3.3. Use in telemonitoring.	NI	PI	PI	NI	PI	PI	PI	PI	NI	NI	PI	PI	PI	NI	NI	NI	NI
	3.4. Confirmed cases registered on the platform and notified in e-SUS Notifica.	I	I	I	I	I	I	I	I	I	I	PI	PI	I	I	I	PI	I
	3.5. Use in contact tracing.	NI	NI	NI	NI	NI	I	PI	NI	NI	NI	NI	NI	I	PI	NI	NI	NI
Dimension 3 status		PI	I	PI	PI	PI	I	PI	PI	PI	PI	PI	PI	PI	PI	PI	PI	PI
Unit status		PI	PI	PI	NI	PI	I	I	PI	PI	PI	PI	PI	PI	PI	PI	PI	PI

UBS: health center; FHU: Family Health Unit; NI: not implemented; PI: partially implemented; I: implemented; NA: not applicable; TQT-Covid: testing, isolation, quarantine and telemonitoring of Covid-19 cases; e-SUS Notifica: online reporting tool for notification of mild flu-like syndrome cases suspected and confirmed of the new coronavirus.

**Chart 3:** Selected respondents’ speeches concerning the analyzed dimensions for assessing the degree of implementation of the TQT-Covid-Strategy.

Dimension 1 – Accessibility to testing
**Health communication strategies** "Any lecture, whatever your intention to carry out an educational activity, is faced with the difficulty of the unit's structure, which is a hallway. So, there's agglomeration. We cannot divide it" (P07; UBS3). "It has even improved it, ‘cause TQT provided and brought materials, pamphlets, posters. We created a lot of waiting rooms here in the unit, delivered pamphlets on the street" (CHA31; FHU1). **Carrying out testing** "When the TQT test was available, it wasn't so crowded to take it, people weren't looking for it that much. So, if the TQT had become available in 2021, we would have better helped the community" (P31; FHU2). "At first, the flow was controlled, but then, when there was that second peak, it got really hard. It was stressful, and once again we had to articulate and divide to organize the waiting line […] as our goal was to provide care as much as possible. There were days we couldn't take care of everyone" (CHA29; FHU11). "I think that it took a little longer with TQT due to that form and many printed questionnaires to fill out. If we could go back, it would be briefer, even for the staff to join the project more effectively" (P22; FHU8).
Dimension 2 – Monitoring of cases and surveillance strategies
**Telemonitoring of positive cases** "Many provided phone numbers didn't work. Because, as it is a violent community, they didn't provide their phone numbers, I mean, one that actually works" (P37; UBS1). "It was properly done, although sometimes some patients did not answer the phone, but all of that was recorded […]. We used our own phones, there's not even a phone here in our unit" (P52; FHU1). "There were only a few problems with the system update. Sometimes there were patients who had already been discharged and continued in the system" (P2; FHU7). **Contact tracing** "So, we did it, but the registration on the platform has been carried out in a way that it seemed like it wasn't, until we could align it with the flow at the meeting […], but in November we couldn't do it, ‘cause we prioritized telemonitoring over contact tracing. We only managed to finish the search for positive patients that was due in November in December" (P17; UBS5). "We also made calls and those who answered it… ‘cause many did not answered it, they would see a private number and decline it. For those who answered it, we asked them to come to the health center to take the Covid test and explained why. Sometimes they came, but sometimes they didn't" (P44; FHU3). "Because the tracing happened in a very superficial way or it didn't happen at all, ‘cause sometimes we would ask the patient in the waiting line: ‘Do you live with someone? Can you inform us your contact details?’. And, you know, sometimes they would say they lived alone, even if they didn't, because when we called them, we knew they lived with someone else" (P20; FHU8). "Because, like, after the vaccines, people would usually say: ‘Oh, I tested positive.’ It was no longer a big deal." (CHA47; FHU11). **Active search for symptomatic individuals in the territory** "Very little has been done, there was hardly that active search you guys have standardized, from the CHA form. But, you know, they [CHAs] said they were providing guidance, but we hardly saw the interview form. If I'm not mistaken, only one health agent did it" (P48; FHU11). "When there was some datum inconsistency […] the nurse would make contact and the community agent would visit the area" (M4; FHU5). "The thing is, first, we did not have enough community agents to encourage the visit to the territory, ‘cause we needed them here to handle the work at the unit" (P8; FHU6).
Dimension 3 - Digital platform
**Use for real-time situation analysis** "When it [TV] was installed, the cases had already dramatically declined." (P28; FHU1). "We had more access when the supervisor came, there was a meeting, she would turn the TV on and show it to us" (CHA42; FHU4). **Use in access to test results** "It happened for a patient or another, I think, there were some technology difficulties. It happens. Sometimes patients did not understand how to access the link sent via WhatsApp, or via text message, and they had difficulties. So I ended up sending them the medical report" (P27; UBS2). **Use in telemonitoring** "The system itself would already schedule it […]. I called, asked, answered the questionnaire, checked how the patient was doing. If necessary, we would provide them with some guidance. If we saw that they needed medical care, we suggested for them to come to the unit. If it were a more severe case, we recommended for them to seek an emergency service" (P3; FHU7). **Percentage of confirmed cases registered on the platform** "At first, there were some difficulties that the facilitator had warned us about, as some data did not appear in the notifications, in the reports, but it was more about technical issues that were promptly resolved when we reached out to them" (P10; FHU6). "That notification thing was excellent. It helped [us] a lot, we notified [patients] much faster" (P12; FHU9).

M: manager; P: professional; CHA: community health agent; FHU: Family Health Unit; UBS: health center; TQT: project of testing, isolation, quarantine, and telemonitoring of Covid-19 cases.

In the component "accessibility to testing," the criteria were assessed as implemented in most units. Several communication strategies were used: distribution of pamphlets; posting of posters; sound trucks; word of mouth; social networks; community radio; activities in waiting room; work in churches, groups of older adults and smoking cessation groups; and activities in schools. We observed greater structuring and organization of testing processes and adoption of communication strategies in the FHUs, probably because these units have more than one team and the presence of CHAs. Despite changes in their attributions during the pandemic, as observed in several countries^22^, the presence of CHAs may also have represented a stronger bond between these teams and the community. Testing was interrupted during workers’ stoppage in some units. Each family health team was responsible for a day of testing at the FHU. During the intervention, there were waiting lines at times when the epidemiological situation worsened with an increase in the number of cases. The completion of the socio-behavioral research questionnaire, technical issues in the digital platform and the Internet connectivity led to delay in the flow of care. With the decentralization of testing to 17 units, there was a reduction in demand in units that had already been carrying out testing, allowing improvements in the organization of the care flow.

Improvements in the infrastructure of the units, including external physical spaces, availability of materials and inputs, and the elaboration of protocols, favored the expansion of testing in the territory. The perceived feasibility achieved^23^ due to the favorable perception of workers and managers in relation to the organization of the testing process may have contributed to the favorable results obtained.

In Brazil, authors of a multicentric investigation carried out with a UBS sample showed a strong PHC response to the pandemic in small municipalities located in rural areas of the Northeast of the country, while those in large urban centers in the Southeast obtained lower scores of an index created to evaluate the performance of PHC^15^. Researchers identified that PHC responded to the pandemic with notification and monitoring of cases, in addition to adopting health education strategies via WhatsApp groups^15^, although risk communication in the territories remained limited^15^.

Regarding the "monitoring of cases and surveillance actions," telemonitoring of cases was considered implemented in seven units (six FHUs and one UBS) and partially implemented in eight (five FHUs and three UBSs). There were reports of noncompliance with the deadlines established during the periods of increase in Covid-19 cases. Overall, this activity was the responsibility of higher-level health professionals (nurses, physicians, and dentists). Despite the statements about the use of scheduling according to the TQT-System, when analyzing the "percentage of telemonitored diagnosed users," in all units the indexes were less than 33.3% (Table 1), being considered as not implemented, demonstrating the difficulty in implementing surveillance actions in the work process within the pandemic context of Covid-19.

The interviewees mentioned difficulties such as technical issues with the platform and the Internet, patients who did not answered phone calls, health units located in places with urban violence with difficulty in reporting the telephone numbers of contacts, wrong telephone numbers, use of the professional's personal mobile phone, and patients without the WhatsApp application. The identification of contacts was deemed not implemented in most units (four UBSs and seven FHUs) and implemented in five (one UBS and four FHUs). Tracing via telephone of the identified contacts was not implemented in 11 units (eight FHUs and three UBSs) and partially implemented in six (four FHUs and two UBSs), with the percentage ranging from 0 to 53.1% (Table 1).

The units reported low user adherence, who did not inform the contacts; furthermore, those registered did not answer the calls or changed the telephone number, probably because the territory was a region of urban violence due to drug trafficking. Noncompliance with the proposed contact tracing strategy^23^ was partly due to the lack of information provided about them. People's support to inform contact history has been highlighted as important in Korea and Vietnam^5^. Technical and data entry issues on the platform were also mentioned, in addition to prioritization of telemonitoring in the peaks of cases. In addition, many contacts did not show up to perform the tests^24^.

The time of the intervention, which coincided with a more favorable epidemiological situation due to vaccination and the relaxation of control measures, may have also contributed to the reduction of adherence, according to the statements. It is also noteworthy that the effectiveness of this action depends on the test-tracing-quarantine process, which requires coordination between the agents involved with surveillance actions, and the studies, whose authors, overall, do not describe the implementation and scope of contact tracing^25^.

The active search for symptomatic individuals in the territory was considered not implemented in 10 FHUs and partially implemented in the remaining two. This criterion was not scored for the UBSs, as it is the responsibility of CHAs. Overall, the search was non-systematic and demand-driven. The insufficient number of CHAs and their need in the unit were mentioned as impediments. Other authors did not observe systematic actions of "active surveillance" to identify respiratory symptoms in the territory^18^. For the active search for symptomatic individuals, the Project proposed the creation of an application with offline operation, but because it was not made available by Google via Playstore, printed questionnaires were chosen, with limited adherence among CHAs.

The digital platform (TQT-System) was considered easy to use, with self-explanatory interface and digitized information; however, technical issues, such as data loss, duplication, and failures, were reported. Tablets were made available for use, but the instability of the Internet in health units made the access difficult. Nonetheless, we observed that the real-time situation analysis tool was not used frequently by the teams, with only one UBS mentioning its constant use. Failure to use the panel to analyze the situation in real time can be related to the moment of flexibilization of social distancing measures in the city and fewer and mild cases after vaccination. Work overload of health professionals and technical issues were also mentioned. Research with emphasis on quality assurance in the use of digital technologies and technical aspects related to digital health infrastructure and platforms is necessary^11^. However, users accessed medical reports with ease in all units.

In turn, the constant use of the digital platform as a whole for telemonitoring was considered as partially implemented in nine units and not implemented in eight. The failure to record telemonitoring activity was related to technical issues of the platform and Internet instability. In terms of contact tracing, in most units, the platform was not used as recommended. Conversely, most of the units (all UBSs and nine FHUs) had notified cases recorded on the platform. The perception of the adequacy of the appropriateness of an innovation for addressing a specific problem appears to favor the implementation^23^, as observed with the use of the platform for case notification. Authors recommend strengthening PHC with the implementation of community surveillance systems and investing in strengthening the digital health system^8^.

### Enablers and Barriers to Implementation

It is worth identifying enablers and barriers for incorporating innovations^16^. Interviewees mentioned the planning of actions of the TQT-Covid-Strategy as a positive factor, encompassing the organization of the work process, standardization of conducts, teamwork, and conflict mediation, as well as the presence of project's professionals for supporting the team. Initially, the physical space of the health units was an obstacle, making it difficult to create a flow for respiratory symptoms. The installation of awnings in the outdoor area partly solved the problem, despite reports about the heat in warmer periods of the day and difficulties when it rained. Moreover, overload in medical agendas during peak of cases, as well as difficulties in organizing shifts due to the lack of professionals with the return of routine activities in PHC, were mentioned.

Overall, implementation strategies, including training, changes in workflow, and distribution of educational materials, favor the incorporation of the proposed innovations^26^. The TQT-Covid-Strategy, in addition to providing protocols, invested in the workforce qualification through a five-month hybrid training program — included in the online course —, with the participation of 353 professionals — 60.7% of the total in the 17 units. There were three face-to-face meetings (175 participants), with presentation of the intervention and planning of monitoring actions and training on procedures for Covid-19 testing for 178 professionals qualified by their class councils (nursing technicians, dentists, nurses, and physicians).

The training process left a legacy of appreciation of workers and prepared the team to cope with new epidemics. Authors of other studies have pointed out advances in the knowledge and experience of professionals in coping with Covid-19; however, they recommend the need for more research on the topic^10^.

A research grant was offered to one professional per health team, unit managers, and CHAs, in such a way that they could lead and act as mediators of the implementation, in addition to systematizing research activities. There was resistance from professionals who did not receive this incentive, which resulted in the reduction of the number of professionals in the shifts in some units. This suggests the need for a greater performance of managers so that everyone understands coping with Covid-19 and health surveillance actions as part of the work of the health unit team.

Conversely, the process of monitoring the implementation of the intervention sought to monitor progress toward implementation and performance goals, identifying challenges, qualifying the adopted strategies, or proposing the introduction of new measures^27^. The solution of problems that arose in the course of the implementation was sought after together with managers and professionals The professionals were overburdened by the return of routine activities, and the project provided listening processes in the adverse scenario. It is also necessary to consider the short time of the intervention that sought a quick response to cope with Covid-19. The adverse scenario made it difficult to incorporate all innovations and strategies in the work process. Integration of real-time implementation tests to adjust and optimize interventions^16^, which takes time, is an approach to be considered in future experiences.

It is noteworthy that qualifying and monitoring the implementation are not enough to change the behavior of PHC workers and incorporate innovations, being important to identify barriers and enablers of acceptance in different contexts and develop implementation strategies that overcome these barriers and strengthen favorable situations^16^. Likewise, the project planning and implementation committee acted on the search for solutions to the identified problems. Researchers emphasize the importance of working in partnership with health managers and professionals, integrating research and practice, aiming at increasing the incorporation of evidence-based innovations in public health^16^.

### Main Results of the TQT-Covid-Strategy According to Professionals

As the main results of the TQT-Covid-Strategy, the interviewed professionals mentioned the expansion of testing in the territory; the identification of cases and the organization of actions; the strengthening of the team-community bond, with humanized care and planned communication; the systematization of information and quick access to results; and the contribution to the institutionalization of surveillance in work processes. Based on the experience and learning, the incorporation of rapid tests for Covid-19 into the Brazilian Unified Health System (SUS), in a permanent and capillarized way in PHC units, was suggested, in addition to the discussion of the organization of the work process and the flow of care, with humanized embracement of users.

Furthermore, the experience of the project and the importance of dialogue before adopting new technologies were also highlighted, in addition to the expansion of the provision of courses and the improvement in health communication and education. For PHC to play its role in responding to health emergencies, it is necessary to strengthen planning, complement budget, expand and qualify the workforce, as well as investments in organizational innovation — as recommended in other studies^7,8,15,28^.

It was emphasized that the necessary conditions should be guaranteed, because the lack of professionals hampered the implementation of telemonitoring, contact tracing, and active search. The low recording of surveillance activities on the digital platform made it difficult to verify to what extent these actions were incorporated into the work processes of the teams. There are, however, some indications that point to the need for reorganization of the work process, aiming at the effective incorporation of surveillance actions into the routine of health services. Other authors highlight the challenge of reorganizing the work processes of the teams aiming at carrying out the actions traditionally developed in surveillance^18,29,30^. The existence of own logics in the different processes would be an obstacle to operationalizing the integration between PHC and health surveillance in the territories^18^.

## Conclusions

Contextual factors (infrastructure, human resources, technical issues of the platform and the Internet connectivity) and structural factors (the integration of surveillance activities into PHC) hampered the implementation of the proposed intervention. The possible lower adherence to some actions may be related to the reduction of popular concern and the flexibilization of Covid-19 control measures at the time the intervention was implemented. Nonetheless, the implementation enabled the expansion of rapid Covid-19 tests (over 13,000 tests), data systematization, and improved the organization of work processes in PHC.

The operational recommendations are directly derived from the enablers and barriers identified during the implementation of the TQT-Covid-Strategy as well as from the lessons learned through practice. The objective is to translate the findings of the evaluation of the degree of implementation (what worked, what did not work, and why) into concrete actions to improve future implementations. This study is limited by not considering the diversity of units (structure, organization, workload, and team) in the score, a factor that may have affected the results. However, the findings suggest structural issues in the organization of the work process.

Preparing PHC for future health crises and ensuring the sustainability and scaling-up of this strategy requires investment in infrastructure, personnel, and training. The qualification of PHC also depends on integrated digital surveillance systems and permanent education. Active engagement of managers and health workers in innovation processes, with adaptations to local contexts, is essential. The implementation should be monitored longitudinally, with continuous adjustments, to maximize the results.

## Data Availability

Research data not included in the article are unavailable.
