# Supplementary material for: Evaluation of the implementation of an intervention aimed at expanding Covid-19 testing, case telemonitoring and surveillance actions in Primary Health Care
Source: Rev Saude Publica. 2026 Apr 17;60(Suppl 1):e13s. doi: 10.11606/s1518-8787.2026060006688 (PMC13089371; doi:10.11606/s1518-8787.2026060006688)
Supplement: Translation

# Translation: Avaliação da implantação de intervenção visando à expansão de testagem para Covid-19, telemonitoramento de casos e ações de vigilância na Atenção Primária à Saúde

**Authors:** Gerluce Alves Pontes da Silva, Thais Regis Aranha Rossi, Sisse Figueiredo de Santana, Alcione Brasileiro Oliveira, Ana Maria Freire de Souza Lima, Trícia Silva Santos, Izabel Cristina Neves Ramos, Carolina Pedroza de Carvalho Garcia, Laio Magno, Inês Dourado, Lígia Maria Vieira da Silva

**Affiliations:** I Universidade Federal da Bahia. Instituto de Saúde Coletiva. Salvador, BA, Brasil; II Universidade do Estado da Bahia. Departamento de Ciências da Vida. Salvador, BA, Brasil; III Universidade Federal da Bahia. Faculdade de Odontologia. Departamento de Odontologia Social e Pediátrica. Salvador, BA, Brasil; IV Secretaria Municipal de Saúde de Camaçari. Camaçari, BA, Brasil; V Fundação Oswaldo Cruz. Instituto Gonçalo Moniz. Salvador, BA, Brasil

**Keywords:** Avaliação em Saúde, Vigilância em Saúde, Atenção Primária à Saúde

## Abstract

**OBJETIVO:**

Avaliar o grau de implantação de uma intervenção que visa expandir testagem, isolamento, quarentena e telemonitoramento de casos de Covid-19 (Estratégia-TQT-Covid) em unidades de atenção primária em uma região administrativa de saúde de um município do Nordeste brasileiro.

**MÉTODOS:**

Utilizou-se uma matriz de julgamento com dimensões e critérios derivados de modelo lógico elaborado com base em revisão de literatura e diretrizes técnicas da Organização Mundial da Saúde, do Ministério da Saúde e do município sede da região administrativa de saúde. Foram realizadas entrevistas com 121 informantes-chave.

**RESULTADOS:**

O grau de implantação nas 17 unidades de saúde estudadas (12 Unidades de Saúde da Família e cinco unidades básicas de saúde) variou em relação a classificação final, dimensões e critérios. Na classificação geral, a maioria das unidades foi considerada com a intervenção parcialmente implantada, sendo a dimensão mais bem classificada "acessibilidade à testagem", implantada em 82,3% das unidades, seguida pelo uso da "plataforma digital", parcialmente implantada em 15 unidades. A dimensão com menor percentual de pontuação foi "acompanhamento de casos e estratégias de vigilância", classificada como não implantada em 76,5% das unidades.

**CONCLUSÃO:**

A intervenção trouxe melhorias importantes no acesso à testagem, com adoção de estratégias de comunicação que favoreceram a adesão da população. Fatores contextuais, operacionais, técnicos e de aceitação pelos profissionais e usuários representaram importantes desafios na implantação da intervenção. Para a sustentabilidade e disseminação dessa estratégia é necessário investir em ações de fortalecimento da Atenção Primária à Saúde, com melhoria da infraestrutura, ampliação da força de trabalho e capacitação dos profissionais.

## INTRODUÇÃO

A pandemia de Covid-19 impôs uma crise sanitária global, gerando desafios complexos para os sistemas de saúde em todo o mundo^1^. A gestão inicial dessa crise evidenciou a dificuldade em aplicar políticas de saúde globais ou nacionais aos contextos locais, muitas vezes com uma ênfase maior na atenção hospitalar em detrimento da vigilância em saúde^2^.

Em resposta, países com experiências exitosas ampliaram o acesso a testes, isolamento, rastreamento de contatos e telemonitoramento^3-6^. A Atenção Primária à Saúde (APS) mostrou ter um papel importante nesse cenário^7-9^, apesar de ter sido impactada significativamente com mudanças relacionadas à organização dos serviços, à saúde dos profissionais, além do formato do atendimento^10-13^. No Brasil, as equipes de APS atuaram fundamentalmente na vigilância e assistência, mesmo enfrentando desafios estruturais^13-15^.

Considerando a necessidade de fortalecer a resposta à pandemia na APS, especialmente em territórios vulneráveis, a Estratégia-TQT-Covid foi proposta com o objetivo de expandir a testagem para Covid-19, o isolamento, a quarentena e o telemonitoramento de casos em unidades de APS. No entanto, a simples existência de uma intervenção não garante sua utilização rotineira; é fundamental compreender como implementá-la, os obstáculos presentes e os fatores facilitadores^16^.

Para o sucesso da implementação de medidas de saúde pública durante a pandemia, a disponibilidade de recursos (equipamentos, pessoal, financeiros) foi essencial^17^. A reorganização dos processos de trabalho das equipes para integrar vigilância e atenção aos casos também representou um desafio^18^. Já a adesão popular atuou como facilitador, impulsionando o sucesso das medidas^5,6^. Para sistematizar as experiências exitosas e os problemas enfrentados, autores destacam a importância de integrar a avaliação no desenho e na implementação de intervenções e políticas de saúde pública^19^. Isso implica a necessidade de construir capacidades específicas para avaliar tais intervenções.

O projeto de pesquisa multicêntrico Estratégia-TQT-Covid foi desenvolvido em uma capital do Nordeste do país. Esta iniciativa contou com o envolvimento de quatro instituições de pesquisa, o apoio do Ministério da Saúde e financiamento de organismo internacional (Unitaid)^20^.

A intervenção foi implantada entre julho de 2022 e fevereiro de 2023, com foco nas dificuldades^21^ identificadas no enfrentamento à pandemia e fundamentada em uma análise de situação: testagem concentrada em poucas unidades de saúde, ausência de identificação precoce de casos e rastreamento de contatantes, ausência ou baixa realização do telemonitoramento dos casos, falta de estratégias de comunicação sobre a Covid-19 nos territórios, dificuldade de acesso aos laudos dos testes, infraestrutura inadequada das unidades básicas de saúde (UBS) e insuficiência de profissionais, dificuldades no registro das ações, falta de dispositivos eletrônicos adequados e conexão de internet instável, preenchimento manual de fichas de notificações, além da falta de oferta de capacitação para os profissionais da APS^21^.

Com o propósito de avaliar a implementação da Estratégia-TQT-Covid, este estudo objetivou determinar o quanto ela foi implantada conforme planejado em unidades de saúde de uma região administrativa específica de um município do Nordeste brasileiro e identificar os elementos que facilitaram ou dificultaram esse processo.

## MÉTODOS

Realizou-se um estudo de avaliação do grau de implantação da Estratégia-TQT-Covid em uma Região Administrativa de Saúde (RAS), com abrangência de cerca de 400 mil habitantes, de um município do Nordeste brasileiro. Foram incluídas todas as UBS de um distrito sanitário. Este distrito foi escolhido por apresentar alta vulnerabilidade social, ter registrado um dos maiores números de casos de Covid-19 no início da pandemia e por ser uma área com forte integração docente-assistencial ligada a uma das instituições de pesquisa colaboradoras.

Adotou-se como critério de seleção das unidades desse distrito a presença de profissionais efetivos em seus quadros, com no mínimo um médico. Assim, foram selecionadas 17 unidades, sendo 12 Unidades de Saúde da Família (USF) e cinco UBS. Criou-se um comitê de planejamento e implementação do projeto com 16 gestores e profissionais da Secretaria Municipal de Saúde (SMS) e da RAS, além de pesquisadores. Esse comitê se reuniu semanalmente e atuou em todo o processo de planejamento e implementação do projeto.

Para desenvolver a pesquisa avaliativa, inicialmente elaborou-se o modelo lógico da intervenção com componentes, ações necessárias, resultados intermediários e finais da intervenção (Figura). Ações para promover a ampliação de testes no território integravam o componente "ampliação da acessibilidade à testagem". Acerca do "acompanhamento dos casos e estratégias de vigilância", foram incluídos meios de prevenção do agravamento dos casos e controle da propagação do vírus. Uma plataforma digital foi estruturada e seu uso teve como objetivo gerenciar as atividades realizadas. Para a construção da modelização, utilizaram-se evidências da literatura científica, protocolos internacionais e nacionais validados pela Organização Mundial da Saúde, pelo Ministério da Saúde e pelo município sede da RAS.

**Figura:** Modelo lógico da Estratégia-TQT-Covid.

No segundo momento, desenvolveu-se uma matriz com dimensões, critérios e padrões de avaliação do grau de implantação da Estratégia-TQT-Covid, submetida a um grupo de 20 especialistas, composto por gestores e profissionais da SMS e da RAS, além de pesquisadores para validação. Buscou-se um consenso em relação aos critérios e padrões propostos.

A versão final da matriz (Quadro 1) apresentou 12 critérios considerados importantes e muito importantes, com médio e alto graus de consenso. Fixou-se a pontuação máxima que a unidade de saúde poderia atingir em 100 pontos, divididos entre as dimensões "acessibilidade à testagem" (40 pontos), "acompanhamento de casos e estratégias de vigilância" (40 pontos) e "plataforma digital" (20 pontos).

**Quadro 1:** Matriz de julgamento para a apreciação do grau de implantação da intervenção TQT-Covid.

Critérios	Padrões para classificação
1.1. Estratégias de comunicação em saúde sobre prevenção e testagem (10 pts.).	1. Não realiza ações de comunicação em saúde em unidades, territórios e redes sociais; 2. Realiza ações esporádicas de comunicação em saúde em unidades, territórios e redes sociais; 3. Realiza ações sistemáticas de comunicação em saúde em unidades, territórios e redes sociais.
1.2. Realização de testagem ampliada e contínua (30 pts.).	1. Presença de interrupções da testagem; 2. Testagem contínua apenas para sintomáticos do território adscrito; 3. Testagem contínua de sintomáticos e contatantes (sintomáticos e assintomáticos) do território adscrito.
2.1. Realização de telemonitoramento dos casos de acordo com o protocolo (5 pts.).	1. Não realiza o telemonitoramento; 2. Realiza, mas não cumpre o prazo estabelecido pelos protocolos municipais; 3. Realiza cumprindo o prazo estabelecido pelos protocolos municipais.
2.2. Percentual de usuários diagnosticados telemonitorados (10 pts.).	1. Não realiza o telemonitoramento; 2. < 80% de casos diagnosticados telemonitorados; 3. > de 80% de casos diagnosticados telemonitorados.
2.3. Identificação de contatantes (5 pts.).	1. Não identifica os contatantes dos casos confirmados; 2. Proporção de contatantes identificados dos casos confirmados < 1; 3. Proporção de contatantes identificados dos casos confirmados > 1.
2.4. Rastreamento de contatantes (10 pts.).	1. Não faz o rastreamento dos contatantes identificados; 2. Faz o contato por telefone ou outros meios de < 80% dos contatantes identificados; 3. Faz o contato por telefone ou outros meios de > 80% dos contatantes identificados.
2.5. Busca ativa de sintomáticos no território (10 pts.).	1. Não realiza; 2. Realiza de forma esporádica; 3. Realiza de forma sistemática.
3.1. Uso para análise de situação em tempo real (4 pts.).	1. Não utiliza a plataforma digital para análise de situação em tempo real; 2. Uso esporádico da plataforma digital para análise de situação em tempo real; 3. Uso sistemático da plataforma digital para análise de situação em tempo real pela equipe.
3.2. Uso no acesso aos resultados de testes (4 pts.).	1. Não utiliza, mesmo com demanda dos usuários por laudo ou para acompanhamento interno da equipe; 2. Uso esporádico, mesmo com demanda dos usuários por laudo ou para acompanhamento interno da equipe; 3. Uso sistemático da plataforma digital para acesso aos resultados de testes por demanda de laudo ou para acompanhamento interno da equipe.
3.3. Uso no telemonitoramento (4 pts.).	1. Não utiliza; 2. Utiliza de forma esporádica para verificar o agendamento e os alertas para o telemonitoramento; 3. Utiliza de forma sistemática para verificar o agendamento e os alertas para o telemonitoramento.
3.4. Percentual de casos confirmados registrados na plataforma e notificados no e-SUS Notifica (4 pts.).	1. Não realiza confirmação de notificações dos casos confirmados; 2. Realiza confirmação de até 80% das notificações dos casos confirmados; 3. Realiza confirmação acima de 80% das notificações dos casos confirmados.
3.5. Uso no rastreamento de contatantes (4 pts.).	1. Não utiliza o módulo de rastreamento para verificação dos contatantes a serem rastreados; 2. Uso esporádico do módulo de rastreamento para verificação dos contatantes a serem rastreados; 3. Uso sistemático do módulo de rastreamento para verificação dos contatantes a serem rastreados.

TQT-Covid: testagem, isolamento, quarentena e telemonitoramento de casos de Covid-19; e-SUS Notifica: ferramenta online de registro de notificação de casos de síndrome gripal leve suspeitos e confirmados do novo coronavírus; pts.: pontos; 1: não implantado; 2: parcialmente implantado; 3: implantado.

A produção de dados abrangeu análise do relatório final do monitoramento da implementação da intervenção; dados do Sistema-TQT-Covid (plataforma digital desenvolvida para a intervenção); e entrevistas semiestruturadas com 121 informantes-chave, envolvendo 15 gerentes, 53 profissionais de saúde e 53 agentes comunitários de saúde (ACS) das unidades selecionadas.

O roteiro das entrevistas foi desenvolvido de acordo com os componentes do modelo lógico. As entrevistas foram realizadas no próprio serviço entre setembro e dezembro de 2022, sendo transcritas e codificadas por meio do software *Nvivo*. A pontuação foi baseada na triangulação das evidências. Isso significa que os avaliadores consideraram e compararam as informações obtidas das diferentes fontes – as entrevistas com os informantes-chave (dados qualitativos e suas interpretações), as informações do relatório de monitoramento e os dados da plataforma digital (dados quantitativos e suas métricas). A triangulação permitiu uma avaliação mais robusta do grau de implantação para cada critério, integrando a percepção e experiência dos profissionais com os dados objetivos de execução.

Para classificar o grau de implantação da Estratégia-TQT-Covid, considerando a matriz de julgamento elaborada, classificou-se o grau de implantação em tercis: não implantada (≥ 0 e < 33,3%), parcialmente implantada (≥ 33,3% e < 66,6%) e implantada (≥ 66,6%). O grau de implantação foi determinado pela porcentagem (%) de pontos obtidos em cada componente relacionado à pontuação máxima proposta. A pesquisa foi aprovada pelo Comitê de Ética em Pesquisa da Organização Mundial da Saúde, sob número de protocolo CERC.0128A e CERC.0128B.

## RESULTADOS e DISCUSSÃO

### Apreciação do Grau de Implantação

O grau de implantação da intervenção nas 17 unidades de saúde variou em relação a classificação final, dimensões e critérios (Quadro 2). Na classificação geral, a maioria das unidades foi considerada com a intervenção parcialmente implantada (dez USF e quatro UBS), sendo a dimensão mais bem classificada a "acessibilidade à testagem", implantada em 82,3% das unidades (12 USF e duas UBS), seguida pelo uso da "plataforma digital", parcialmente implantada em 15 unidades (11 USF e quatro UBS). A dimensão "acompanhamento de casos e estratégias de vigilância" obteve a menor pontuação, classificada como não implantada em 76,5% das unidades (nove USF e quatro UBS). Sínteses das evidências são apresentadas na Tabela e Quadro 3.

**Tabela:** Indicadores da matriz de julgamento para apreciação do grau de implantação da intervenção TQT-Covid em uma região administrativa de saúde em um município do Nordeste brasileiro (variação de julho de 2022 a março de 2023).

Unidade	Percentual de usuários diagnosticados telemonitorados		Proporção de contatantes identificados em relação aos usuários com TR+		Percentual de contatantes rastreados encontrados		Percentual de casos confirmados registrados na plataforma e notificados no e-SUS Notifica		
	N.º de usuários TR+ telemonitorados (≥ 1 contato)	% de usuários TR+ com ≥ 1 contato	N.º de contatantes identificados	Contatantes identificados/usuários TR+	N.º de contatantes rastreados encontrados	% de contatantes rastreados encontrados entre os identificados	N.º de usuários com TR+	N.º de casos TR+ registrados na plataforma com ID do e-SUS Notifica	%
USF 1	37	19,8	160	0,85	85	53,1	187	187	100
USF 2	23	26,1	95	1,08	42	44,2	88	83	94,0
USF 3	29	32,2	12	0,13	0	0	90	87	96,7
USF 4	10	8,2	7	0,06	1	14,3	122	119	97,5
USF 5	10	12,3	15	0,18	0	0	81	78	96,3
USF 6	41	12,2	33	0,10	12	36,4	337	158	46,8
USF 7	42	21,9	34	0,18	7	20,6	192	106	55,2
USF 8	10	11,6	61	0,70	22	36,1	86	86	100
USF 9	0	0	207	0,96	0	0	216	216	100
USF 10	3	2,7	40	0,36	3	7,5	112	110	98,2
USF 11	3	2,4	3	0,02	0	0	124	101	81,4
USF 12	1	1,4	57	0,77	0	0	74	73	98,6
Valor total USF	209	12,2	724	0,42	172	23,7	1.709	1.404	82,1
UBS 1	5	13,2	5	0,13	1	20,0	38	32	84,0
UBS 2	8	17,4	3	0,06	0	0	46	43	93,5
UBS 3	49	16,4	14	0,05	5	35,7	298	298	100
UBS 4	2	2,9	1	0,01	0	0	70	70	100
UBS 5	49	31,6	110	0,71	53	48,2	155	150	96,8
Valor total UBS	113	18,6	133	0,22	59	44,4	607	593	97,7
Valor total unidades	322	13,9	857	0,37	231	27,0	2.316	1.997	86,2

TR+: teste positivo; USF: Unidade de Saúde da Família; UBS: unidade básica de saúde; ID do e-SUS Notifica: número de identificação da notificação no sistema.

**Quadro 2:** Classificação do grau de implantação da intervenção TQT-Covid por Unidade Básica de Saúde em região administrativa de município do Nordeste.

Dimensão	Critério	Situação de implantação por unidade																
		UBS 1	UBS 2	UBS 3	UBS 4	UBS 5	USF 1	USF 2	USF 3	USF 4	USF 5	USF 6	USF 7	USF 8	USF 9	USF 10	USF 11	USF 12
1. Acessibilidade à testagem	1.1. Estratégias de comunicação sobre prevenção e testagem.	I	PI	PI	NI	PI	I	I	I	I	I	I	I	I	I	I	PI	PI
	1.2. Realização de testagem ampliada e contínua.	PI	PI	I	PI	I	I	I	I	I	I	I	I	PI	I	I	I	I
Situação da Dimensão 1		I	PI	I	PI	I	I	I	I	I	I	I	I	PI	I	I	I	I
2. Acompanhamento de casos e estratégias de vigilância	2.1. Telemonitoramento dos casos positivos de acordo com o protocolo.	PI	I	PI	NI	PI	I	PI	I	I	I	PI	I	I	NI	PI	PI	PI
	2.2. Percentual de usuários diagnosticados telemonitorados.	NI	NI	NI	NI	NI	NI	NI	NI	NI	NI	NI	NI	NI	NI	NI	NI	NI
	2.3. Identificação de contatantes.	NI	NI	NI	NI	I	I	I	NI	NI	NI	NI	NI	PI	I	NI	NI	I
	2.4. Rastreamento de contatantes.	NI	NI	PI	NI	PI	PI	PI	NI	NI	NI	PI	NI	PI	NI	NI	NI	NI
	2.5. Busca ativa de sintomáticos no território.	NA	NA	NA	NA	NA	NI	PI	NI	PI	NI	NI	NI	NI	NI	NI	NI	NI
Situação da Dimensão 2		NI	NI	NI	NI	PI	PI	PI	NI	NI	NI	NI	NI	PI	NI	NI	NI	NI
3. Plataforma digital	3.1. Uso para análise de situação em tempo real.	NI	I	NI	NI	NI	NI	NI	NI	NI	PI	PI	NI	NI	NI	PI	NI	NI
	3.2. Uso no acesso aos resultados de teste.	I	I	I	I	I	I	I	I	I	I	I	I	I	I	I	I	I
	3.3. Uso no telemonitoramento.	NI	PI	PI	NI	PI	PI	PI	PI	NI	NI	PI	PI	PI	NI	NI	NI	NI
	3.4. Casos confirmados registrados na plataforma e notificados no e-SUS Notifica.	I	I	I	I	I	I	I	I	I	I	PI	PI	I	I	I	PI	I
	3.5. Uso no rastreamento de contatantes.	NI	NI	NI	NI	NI	I	PI	NI	NI	NI	NI	NI	I	PI	NI	NI	NI
Situação da Dimensão 3		PI	I	PI	PI	PI	I	PI	PI	PI	PI	PI	PI	PI	PI	PI	PI	PI
Situação da unidade		PI	PI	PI	NI	PI	I	I	PI	PI	PI	PI	PI	PI	PI	PI	PI	PI

UBS: unidade básica de saúde; USF: Unidade Saúde da Família; NI: não implantado; PI: parcialmente implantado; I: implantado; NA: não se aplica; TQT-Covid: testagem, isolamento, quarentena e telemonitoramento de casos de Covid-19; e-SUS Notifica: ferramenta online de registro de notificação de casos de síndrome gripal leve suspeitos e confirmados do novo coronavírus.

**Quadro 3:** Falas selecionadas de entrevistados relacionadas às dimensões analisadas para apreciação do grau de implantação da Estratégia-TQT-Covid.

Dimensão 1 – Acessibilidade à testagem
**Estratégias de comunicação em saúde** "Qualquer palestra, qualquer intenção que você tenha de fazer uma atividade educativa, se depara na dificuldade do formato da unidade, que é um corredor. Então, cria uma aglomeração. A gente não tem como separar" (P07; UBS3). "Até melhorou, porque o TQT ofereceu, trouxe materiais, panfleto, cartazes. A gente fez bastante sala de esperas aqui na unidade, fez panfletagem na rua" (ACS31; USF1). **Realização de testagem** "Quando o teste do TQT chegou, já não estava tão cheio de gente para fazer teste, já procurava menos. Então, o TQT, se ele tivesse chegado em 2021, a gente tinha ajudado melhor a comunidade" (P31; USF2). "No início era controlado [o fluxo], mas depois, quando houve aquele segundo pico, a coisa extrapolou. Era estresse, e mais uma vez a gente tinha que se articular e dividir para organizar a fila […] porque o objetivo da gente era acolher o máximo possível. Tinha dia que não dava conta de todos" (ACS29; USF11). "Eu acho que com o TQT demorou mais um pouco, por causa daquele formulário gigantesco e muitos impressos para preencher. Se pudesse voltar atrás, seria uma coisa mais sucinta, até para o pessoal aderir mais ao projeto" (P22; USF8).
Dimensão 2 – Acompanhamento de casos e estratégias de vigilância
**Realização de telemonitoramento dos casos positivos** "Muitos telefones fornecidos não funcionavam. Porque, por ser uma comunidade de risco, então eles não davam o telefone, assim, que funcione" (P37; UBS1). "Era feito direitinho, apesar de que alguns pacientes às vezes não atendiam o telefone, mas isso era tudo registrado […]. Era o nosso telefone pessoal, aqui na nossa unidade nem telefone tem" (P52; USF1). "Só houve alguns probleminhas com relação à atualização do sistema. Às vezes tinha paciente que já tinha recebido alta e continuava no sistema" (P2; USF7). **Rastreamento de contatantes** "Então a gente fazia, mas o cadastro na plataforma estava sendo feito de uma forma que parecia que não estava sendo feito, até que a gente conseguia alinhar isso no fluxo na reunião […], mas no mês de novembro não foi possível, porque a gente priorizou telemonitorar do que rastrear os contactantes e a gente só conseguiu finalizar a busca dos pacientes positivos do mês de novembro no mês de dezembro" (P17; UBS5). "A gente ligava também e quem atendia… porque muitos não atendiam; via número privado e não atendia. Quem atendia, a gente solicitava que viesse para o posto para fazer o teste da Covid e explicava o motivo. Às vezes vinha, mas às vezes não vinha" (P44; USF3). "Porque o rastreamento aconteceu muito superficial ou não aconteceu, porque às vezes você pergunta ao paciente na fila: ‘Você mora com alguém? Você pode dar os dados do seu contactante?’. E, tipo assim, às vezes ele dizia que não morava com ninguém, mesmo morando, porque quando a gente liga, ele mora com alguém" (P20; USF8). "Porque, assim, depois das vacinas, se tornou um pouco normal a pessoa: ‘Ah, estou positivo’. Não era mais tão assustador assim." (ACS47; USF11). B**usca ativa de sintomáticos no território** "Muito pouco foi feito, quase não teve aquela busca ativa que vocês padronizaram, do formulário do ACS. Mas, assim, eles [os ACS] falavam que orientavam, mas de trazer a ficha com a entrevista foi muito pouco mesmo. Se eu não me engano, só um agente de saúde fez" (P48; USF11). "Quando tinha uma inconsistência de algum dado […] a enfermeira contactava e o agente comunitário ia na área" (G4; USF5). "A questão, primeiro, é que a gente não tinha um quantitativo de agentes comunitários suficiente para incentivar a ida ao território, porque a gente precisava deles aqui para dar conta de todos os serviços da unidade" (P8; USF6).
Dimensão 3 - Plataforma digital
**Uso para análise de situação em tempo real** "Quando ela [TV] foi instalada, os casos já tinham diminuído drasticamente." (P28; USF1). "A gente tinha mais acesso quando chegava a supervisora, tinha reunião, ela ligava a televisão e mostrava" (ACS42; USF4). **Uso no acesso aos resultados de testes** "Acontecia de um paciente ou outro, eu acho, que tinha dificuldade de tecnologia. Acontece. E às vezes o paciente não entendia como é que ele fazia para pegar aquele link que vocês mandavam via WhatsApp, ou via mensagem de texto, e eles tinham dificuldade. E eu acabava passando o laudo" (P27; UBS2). **Uso no telemonitoramento** "O sistema mesmo já agendava […]. Eu ligava, perguntava, respondia o questionário, como é que o paciente estava. Se ele precisasse de alguma orientação, a gente dava. Se a gente via que ele precisava de um atendimento médico, sugeria que ele viesse para unidade. Se fosse um caso mais grave, dirigisse até uma unidade de pronto atendimento" (P3; USF7). Percentual de casos confirmados registrados na plataforma "No início, ela teve algumas dificuldades que a facilitadora comunicou porque alguns dados não apareceram nas notificações, nos relatórios, mas eram mais questões técnicas que quando a gente entrava em contato prontamente era resolvido" (P10; USF6). "Uma coisa excelente foi aquela questão da notificação. Ajudou muito, a gente fez a notificação muito mais rápido" (P12; USF9).

G: gestor; P: profissional; ACS: agente comunitário de saúde; USF: Unidade de Saúde da Família; UBS: unidade básica de saúde; TQT: Projeto de testagem, isolamento, quarentena e telemonitoramento de casos de Covid-19.

No componente "ampliação da acessibilidade à testagem", os critérios foram apreciados como implantados na maioria das unidades. Diversas estratégias de comunicação foram utilizadas: distribuição de folders; fixação de cartazes; carro de som; divulgação "boca a boca"; redes sociais; rádio comunitária; sala de espera; trabalho em igrejas, grupos de idosos e de tabagismo; e atividades nas escolas. Observou-se uma maior estruturação e organização dos processos de testagem e adoção de estratégias de comunicação nas USF, provavelmente por essas unidades terem mais de uma equipe e pela presença dos ACS. Apesar de mudanças em suas atribuições durante a pandemia, como observado em diversos países^22^, a presença dos ACS pode ter representado também um maior vínculo dessas equipes com a comunidade. Houve interrupção da testagem durante paralisações de servidores em algumas unidades. Cada equipe de saúde da família era responsável por um dia de testagem nas USF. Durante a intervenção, houve filas em momentos em que a situação epidemiológica se agravou com aumento do número de casos. O preenchimento do questionário sociocomportamental da pesquisa realizada e problemas técnicos na plataforma digital e de internet causaram demora no fluxo de atendimento. Com a descentralização da realização de testes para 17 unidades, houve redução da demanda nas unidades que já realizavam testagem, permitindo melhoria na organização do fluxo.

As melhorias na infraestrutura das unidades, abrangendo o espaço físico externo, a disponibilização de materiais e insumos e a elaboração de protocolos, favoreceram a ampliação da testagem no território. A viabilidade construída^23^ devido à percepção favorável dos trabalhadores e gerentes em relação à organização do processo de testagem possivelmente contribuiu com os resultados favoráveis obtidos.

No Brasil, uma investigação multicêntrica realizada por meio de amostra de UBS evidenciou boa resposta da APS à pandemia em municípios de pequeno porte situados em áreas rurais do Nordeste do país, enquanto aqueles nos grandes centros urbanos no Sudeste obtiveram um *score* menor de um índice criado para avaliar o desempenho da APS^15^. Esse estudo identificou que a APS respondeu à pandemia com a notificação e o monitoramento de casos, além da adoção de estratégias de educação em saúde por meio dos grupos de WhatsApp^15^, com limitação da comunicação dos riscos nos territórios^15^.

Em relação ao "acompanhamento dos casos e ações de vigilância", a realização de telemonitoramento dos casos foi considerada implantada em sete unidades (seis USF e uma UBS) e parcialmente implantada em oito (cinco USF e três UBS). Houve relatos de não cumprimento dos prazos estabelecidos nos períodos de aumento de casos por Covid-19. No geral, essa atividade era de responsabilidade de profissionais de nível superior (enfermeiros, médicos e dentistas). Apesar dos depoimentos sobre o uso do agendamento de acordo com o Sistema-TQT, ao se analisar o "percentual de usuários diagnosticados e telemonitorados", em todas as unidades os índices foram menores que 33,3% (Tabela), sendo considerado como não implantado, o que revela dificuldade de implementação das ações de vigilância no processo de trabalho no cenário pandêmico da Covid-19.

Os entrevistados citaram dificuldades como problemas técnicos da plataforma e de internet, pacientes que não atendiam às ligações, unidades de saúde em locais de violência urbana com dificuldade de relato dos números de telefone dos contatantes, números de telefone errados, uso do celular do próprio profissional e pacientes sem o aplicativo WhatsApp*.* A identificação de contatantes foi considerada não implantada na maioria das unidades (quatro UBS e sete USF) e implantada em cinco (uma UBS e quatro USF). O rastreamento por telefone dos contatantes identificados não foi implantado em 11 unidades (oito USF e três UBS) e parcialmente implantado em seis (quatro USF e duas UBS), com o percentual variando de 0 a 53,1% (Tabela).

As unidades relataram baixa adesão dos usuários, que não informavam os contatantes, além de que os registrados não atendiam às ligações ou mudavam o número do telefone, provavelmente em virtude de o território ser uma região de violência urbana devido ao tráfico. A não fidelidade ao proposto^23^ no rastreamento de contatantes decorreu, em parte, da falta de informações prestadas sobre eles. O apoio das pessoas para informar o histórico de contatos foi apontado como importante na Coreia e no Vietnã^5^. Problemas técnicos e de registro na plataforma também foram mencionados, além da priorização do telemonitoramento nos picos de casos. Ainda, muitos contatados não compareciam para realizar os testes^24^.

O momento da intervenção, em situação epidemiológica menos desfavorável devido à vacinação e com flexibilização das medidas de controle, possivelmente também contribuiu para a diminuição da adesão, segundo os relatos. Ressalta-se, ainda, que a efetividade dessa ação depende do processo teste-rastreamento-quarentena, que requer coordenação entre os agentes envolvidos com as ações de vigilância, com os estudos, que em geral não descrevem a implementação do rastreamento de contatos e a cobertura desse rastreio^25^.

A busca ativa de sintomáticos no território foi considerada não implantada em dez USF e parcialmente implantada em outras duas. Esse critério não foi pontuado para as UBS, por configurar atividade dos ACS. No geral, a busca realizada era não sistemática, atendendo demandas. O número insuficiente de ACS e sua necessidade na unidade foram impedimentos apontados. Outro estudo não observou ações sistemáticas de "vigilância ativa" para identificação de sintomáticos respiratórios no território^18^. Para a busca ativa de sintomáticos, o Projeto propôs a criação de um aplicativo com funcionamento offline, mas por não ter sido disponibilizado pelo Google na *Playstore*, optou-se por questionários impressos, com pouca adesão dos ACS.

A plataforma digital (Sistema-TQT) foi considerada de fácil uso, com interface autoexplicativa e informações digitalizadas; no entanto, problemas técnicos como perda, duplicação e falhas nos dados foram relatados. Tablets foram disponibilizados para seu uso, mas a instabilidade da internet nas unidades de saúde dificultava o acesso. Observou-se, contudo, que sua ferramenta de análise da situação em tempo real foi pouco utilizada pelas equipes, só mencionada com uso constante em uma UBS. O não uso do painel para análise da situação em tempo real pode ser relacionado ao momento de flexibilização das medidas de distanciamento físico no município e diante de casos em menor número e sem gravidade, após a vacinação. Também foram citados a sobrecarga de trabalho dos profissionais de saúde e problemas técnicos. Pesquisas com ênfase na garantia de qualidade no uso das tecnologias digitais e em aspectos técnicos referentes a infraestrutura e plataformas digitais de saúde são necessárias^11^. No entanto, em todas as unidades, os usuários acessaram os laudos com facilidade.

Já o uso constante da totalidade da plataforma digital no telemonitoramento foi apreciado como parcialmente implantado em nove unidades e não implantado em oito unidades. O não registro da atividade de telemonitoramento foi relacionado a problemas técnicos da plataforma e instabilidade na internet. Para o rastreamento de contatantes, na maioria das unidades, a plataforma não foi utilizada como preconizado. Por outro lado, grande parte das unidades (todas as UBS e nove USF) teve o registro de casos notificados na plataforma. A percepção sobre a adequação da inovação para resolver uma questão específica parece favorecer a implementação^23^, como ocorreu com o uso da plataforma para notificação de casos. Autores recomendam o fortalecimento da APS com a implementação de sistemas de vigilância comunitária e o investimento no fortalecimento do sistema de saúde digital^8^.

### Fatores Facilitadores e Barreiras para a Implantação

É importante identificar fatores facilitadores e barreiras para a incorporação de inovações^16^. Entrevistados mencionaram o planejamento das ações da Estratégia-TQT-Covid como um fator positivo, abrangendo a organização do processo de trabalho, a uniformização das condutas, o trabalho em equipe e a mediação de conflitos, como também a presença de profissionais do projeto como suporte à equipe. Inicialmente, o espaço físico das unidades de saúde foi um obstáculo, dificultando a criação de fluxo para sintomáticos respiratórios. A instalação de toldos na área externa resolveu parte do problema, apesar dos relatos sobre o calor nos períodos mais quentes do dia e dificuldades em caso de chuvas. Cita-se também sobrecarga nas agendas médicas durante picos de casos, além de dificuldades para organizar as escalas devido à falta de profissionais, com o retorno das atividades de rotina na APS.

No geral, estratégias de implementação, entre elas treinamentos, mudanças no fluxo de trabalho e distribuição de materiais educativos, favorecem a incorporação de inovações propostas^26^. A Estratégia-TQT-Covid, além da disponibilização de protocolos, investiu na formação dos trabalhadores, desenvolvida de forma híbrida durante cinco meses — parte do curso na modalidade online —, com participação de 353 profissionais — 60,7% do total de profissionais das 17 unidades. Foram três encontros presenciais (175 participantes), com apresentação da intervenção e pactuação das ações de monitoramento e uma capacitação sobre os procedimentos de testagem para Covid-19 para 178 profissionais habilitados pelos seus conselhos de classe (técnicos de enfermagem, cirurgiões-dentistas, enfermeiros e médicos).

O processo de formação deixou um legado de valorização dos trabalhadores e preparou a equipe para enfrentamento de novas epidemias. Outros estudos apontaram avanços no conhecimento e na experiência dos profissionais no enfrentamento à Covid-19, no entanto, recomendam a necessidade de mais pesquisas sobre o tema^10^.

Foi ofertada uma bolsa de pesquisa para um profissional por equipe de saúde, gerentes das unidades e ACS, no intuito de que estes liderassem e atuassem como mediadores da implementação, além de sistematização das atividades de pesquisa. Houve resistência de profissionais que não recebiam esse incentivo, o que resultou na redução do número de profissionais nas escalas em algumas unidades. Isso indica a necessidade de uma maior atuação dos gestores para que todos entendam o enfrentamento à Covid-19 e as ações de vigilância à saúde como parte do trabalho da equipe da unidade de saúde.

Por outro lado, o processo de acompanhamento da implantação da intervenção buscou monitorar o progresso em direção às metas de implementação e de desempenho, identificando-se desafios, qualificando as estratégias adotadas ou propondo-se a introdução de novas medidas^27^. Buscou-se, junto aos gestores e profissionais, a solução dos problemas que surgiram no curso da implementação. Os profissionais estavam sobrecarregados com o retorno das atividades de rotina, e o projeto abriu processos de escuta no cenário adverso. Tem-se, ainda, que se considerar o tempo curto da intervenção que buscava uma resposta rápida para enfrentamento da Covid-19. O cenário adverso dificultou a incorporação de todas as inovações e estratégias no processo de trabalho. A integração de testes de implementação em tempo real para ajustar e otimizar as intervenções^16^, o que demanda tempo, pode ser uma abordagem a ser considerada em experiências futuras.

Ressalta-se que capacitar e monitorar a implementação não são suficientes para mudar o comportamento dos trabalhadores da APS e incorporar inovações, sendo importante identificar barreiras e facilitadores de aceitação em diferentes contextos e desenvolver estratégias de implementação que superem essas barreiras e fortaleçam as situações favoráveis^16^. Nessa direção, o comitê de planejamento e implementação do projeto atuou na busca de soluções para os problemas identificados. Outros autores enfatizam a importância de se trabalhar em parceria com gestores e profissionais de saúde, integrando a pesquisa e a prática, visando ao aumento da incorporação de inovações baseadas em evidências na saúde pública^16^.

### Principais Resultados da Estratégia-TQT-Covid na Percepção dos Profissionais

Os profissionais entrevistados identificaram como principais resultados da Estratégia-TQT-Covid a ampliação da testagem no território; a identificação de casos e o direcionamento das ações; o fortalecimento do vínculo equipe-comunidade, com acolhimento humanizado e comunicação planejada; a sistematização das informações e o acesso rápido aos resultados; e a contribuição para a institucionalização da vigilância nos processos de trabalho. Com base na experiência e no aprendizado, foi sugerida a incorporação no Sistema Único de Saúde (SUS) de testes rápidos para Covid-19 de forma permanente e capilarizada nas unidades de APS, além da discussão da organização do processo de trabalho e do fluxo de atendimento, com acolhimento humanizado dos usuários.

Destacaram-se, ainda, a experiência do projeto e a importância do diálogo antes da adoção de novas tecnologias, além da ampliação da oferta de cursos e da melhoria na comunicação e educação em saúde. Para a APS desempenhar seu papel na resposta a emergências sanitárias, torna-se necessário o fortalecimento do planejamento, reforço do orçamento, a ampliação e qualificação da força de trabalho, além de investimentos em inovação organizacional — como recomendado em outros estudos^7,8,15,28^.

Enfatizou-se que as condições necessárias devem ser garantidas, pois a falta de profissionais dificultou a implementação do telemonitoramento, o rastreamento de contatantes e a busca ativa. O baixo registro de atividades de vigilância na plataforma digital dificultou verificar em que medida essas ações foram incorporadas aos processos de trabalho das equipes. Há, contudo, algumas indicações que apontam para a necessidade de reorganização do processo de trabalho, visando a efetiva incorporação das ações de vigilância na rotina dos serviços de saúde. Outros estudos destacam o desafio da reorganização dos processos de trabalho das equipes objetivando a execução das ações desenvolvidas tradicionalmente nas vigilâncias^18,29,30^. A existência de lógicas próprias nos diferentes processos seria obstáculo para operacionalizar a integração entre APS e vigilância em saúde nos territórios^18^.

## CONCLUSÃO

Fatores contextuais (infraestrutura, recursos humanos, problemas técnicos da plataforma e de internet) e estruturais (integração da vigilância na APS) dificultaram a implantação plena da intervenção proposta. A possível menor adesão a algumas ações se relaciona à redução da preocupação popular e à flexibilização dos controles da Covid-19 no momento em que a intervenção foi implementada. Mesmo assim, esta ampliou testes rápidos de Covid-19 (> 13 mil), sistematizou dados e organizou o trabalho na APS.

As recomendações operacionais derivam diretamente dos fatores facilitadores e das barreiras identificadas durante a implantação da Estratégia-TQT-Covid, bem como das lições aprendidas na prática. O objetivo é traduzir os resultados da avaliação do grau de implantação (o que funcionou, o que não funcionou e por quê) em ações concretas para melhorar futuras implementações. Este estudo limita-se por não considerar a diversidade das unidades (estrutura, organização, carga horária e equipe) na pontuação, fator que pode ter afetado os resultados. No entanto, os achados sugerem problemas estruturais na organização do processo de trabalho.

Preparar a APS para novas crises e sustentar/disseminar essa estratégia exige investimento em infraestrutura, pessoal e capacitação. A qualificação da APS também depende de sistemas digitais de vigilância integrados e educação permanente. É crucial envolver gestores e trabalhadores nas inovações, com adaptação ao contexto local. A implementação deve ser acompanhada longitudinalmente, com ajustes contínuos, para maximizar resultados.

## Disponibilidade de Dados:

Os dados da pesquisa não inclusos no artigo não estão disponíveis.
